# Comparative analysis of dynamic transcriptomes reveals specific COVID-19 features and pathogenesis of immunocompromised populations

**DOI:** 10.1128/msystems.01385-23

**Published:** 2024-05-16

**Authors:** Xiaodi Yang, Jialin Zhu, Qingyun Wang, Bo Tang, Ye Shen, Bingjie Wang, Li Ji, Huihui Liu, Stefan Wuchty, Ziding Zhang, Yujun Dong, Zeyin Liang

**Affiliations:** 1 Department of Hematology, Peking University First Hospital, Beijing, China; 2 Department of Computer Science, University of Miami, Miami, Florida, USA; 3 Department of Biology, University of Miami, Miami, Florida, USA; 4 Institute of Data Science and Computation, University of Miami, Miami, Florida, USA; 5 Sylvester Comprehensive Cancer Center, University of Miami, Miami, Florida, USA; 6 College of Biological Sciences, China Agricultural University, Beijing, China; Pacific Northwest National Laboratory, Richland, Washington, USA

**Keywords:** SARS-CoV-2, infection, transcriptome, protein-protein interaction, protein complex, hematological malignancies

## Abstract

A dysfunction of human host genes and proteins in coronavirus infectious disease 2019 (COVID-19) caused by severe acute respiratory syndrome coronavirus 2 (SARS-CoV-2) is a key factor impacting clinical symptoms and outcomes. Yet, a detailed understanding of human host immune responses is still incomplete. Here, we applied RNA sequencing to 94 samples of COVID-19 patients with and without hematological tumors as well as COVID-19 uninfected non-tumor individuals to obtain a comprehensive transcriptome landscape of both hematological tumor patients and non-tumor individuals. In our analysis, we further accounted for the human-SARS-CoV-2 protein interactome, human protein interactome, and human protein complex subnetworks to understand the mechanisms of SARS-CoV-2 infection and host immune responses. Our data sets enabled us to identify important SARS-CoV-2 (non-)targeted differentially expressed genes and complexes post-SARS-CoV-2 infection in both hematological tumor and non-tumor individuals. We found several unique differentially expressed genes, complexes, and functions/pathways such as blood coagulation (APOE, SERPINE1, SERPINE2, and TFPI), lipoprotein particle remodeling (APOC2, APOE, and CETP), and pro-B cell differentiation (IGHM, VPREB1, and IGLL1) during COVID-19 infection in patients with hematological tumors. In particular, APOE, a gene that is associated with both blood coagulation and lipoprotein particle remodeling, is not only upregulated in hematological tumor patients post-SARS-CoV-2 infection but also significantly expressed in acute dead patients with hematological tumors, providing clues for the design of future therapeutic strategies specifically targeting COVID-19 in patients with hematological tumors. Our data provide a rich resource for understanding the specific pathogenesis of COVID-19 in immunocompromised patients, such as those with hematological malignancies, and developing effective therapeutics for COVID-19.

**IMPORTANCE:**

A majority of previous studies focused on the characterization of coronavirus infectious disease 2019 (COVID-19) disease severity in people with normal immunity, while the characterization of COVID-19 in immunocompromised populations is still limited. Our study profiles changes in the transcriptome landscape post-severe acute respiratory syndrome coronavirus 2 (SARS-CoV-2) infection in hematological tumor patients and non-tumor individuals. Furthermore, our integrative and comparative systems biology analysis of the interactome, complexome, and transcriptome provides new insights into the tumor-specific pathogenesis of COVID-19. Our findings confirm that SARS-CoV-2 potentially tends to target more non-functional host proteins to indirectly affect host immune responses in hematological tumor patients. The identified unique genes, complexes, functions/pathways, and expression patterns post-SARS-CoV-2 infection in patients with hematological tumors increase our understanding of how SARS-CoV-2 manipulates the host molecular mechanism. Our observed differential genes/complexes and clinical indicators of normal/long infection and deceased COVID-19 patients provide clues for understanding the mechanism of COVID-19 progression in hematological tumors. Finally, our study provides an important data resource that supports the increasing value of the application of publicly accessible data sets to public health.

## INTRODUCTION

According to the statistics of the World Health Organization from October 2023, the coronavirus infectious disease 2019 (COVID-19), which is caused by the severe acute respiratory syndrome coronavirus 2 (SARS-CoV-2), has led to over 760 million cases and 6.9 million deaths since December 2019 worldwide. Notably, COVID-19 infections show remarkable differences in symptoms in populations where several high-risk factors, such as advanced age, basic diseases, and deficient immune systems, have led to a poor prognosis. In particular, immunocompromised populations, such as patients with hematological malignancies, have weaker immune responses to SARS-CoV-2 infection and virus clearance capacities, contributing to adverse outcomes and significantly higher fatalities (1). As COVID-19 can cause acute mortality and persistent infection in such patients (2), a thorough understanding of the underlying pathogenic mechanisms is of utmost importance to control the pandemic, which has a profound clinical impact on vulnerable immunocompromised populations. Multiple studies have demonstrated the alterations in host immune responses as one of the key factors that led to severe clinical symptoms and outcomes (3–6). While recent studies have provided deeper insights, a comprehensive comparative transcriptome and interactome analysis of SARS-CoV-2 infection and the host immune response of COVID-19 patients with hematological tumors and non-tumor individuals with COVID-19 is still missing, which allows us to dissect the hematological tumor-specific pathogenesis of COVID-19 and illustrate the potential difference in clinical outcomes.

RNA sequencing and interactome analysis are powerful techniques to reveal the mechanisms of viral infection/host immune responses in the presence of COVID-19 infections (7–12). While previous studies of COVID-19 have provided important molecular mechanistic insights, a systematic comparison of transcriptomes of COVID-19 patients with and without hematological tumors or hematological tumor patients with different infection statuses according to clinical outcomes is still missing. Here, we obtained RNA-Seq data from a cohort of 94 samples, including hospitalized hematological tumor patients with COVID-19 and non-tumor individuals with COVID-19 as well as healthy uninfected individuals. As viruses usually take advantage of cellular functions to complete their life cycle through molecular interactions with human host proteins, an understanding of the human-SARS-CoV-2 protein interactome is also critical for our knowledge of the pathogenesis of COVID-19 in the presence of hematological tumors. While a substantial amount of protein interactions between the human host and SARS-CoV-2 proteins have been determined (13–17), such studies usually ignore that viral infection is tightly linked to host protein complexes (18, 19), which allow viruses to tap host cellular processes (20), prompting us to integrate human-SARS-CoV-2 interaction and human protein complex data and analyze SARS-CoV-2 infection and host immune processes on an interactome and protein complex level.

Our integrated analysis of the interactome, complexome, and transcriptome allowed us to show that SARS-CoV-2 tended to target more non-functional host proteins (such as non-essential genes and non-host factors) to indirectly affect host immune functions in hematological tumor patients compared to non-tumor individuals. We also observed several unique key genes, functions/pathways, and complexes in hematological tumor patients, such as blood coagulation (APOE, SERPINE1, SERPINE2, and TFPI; Factor-Xa-TFPI-factor-Vila-tissue factor complex), very-low-density lipoprotein particle remodeling (APOC2, APOE, and CETP), and pro-B cell differentiation (IGHM-VPREB1-IGLL1 complex), which were affected by SARS-CoV-2 infection. Furthermore, our data provide a resource to elucidate the characteristics of gene expression and host immune differences between hematological tumor patients suffering from SARS-CoV-2 infection with different clinical outcomes (i.e., normal infection, long infection, and acute death). Our data and findings may offer important implications for our mechanistic understanding of COVID-19 in patients with hematological tumors.

## MATERIALS AND METHODS

### Biological samples

**Fig 1:**
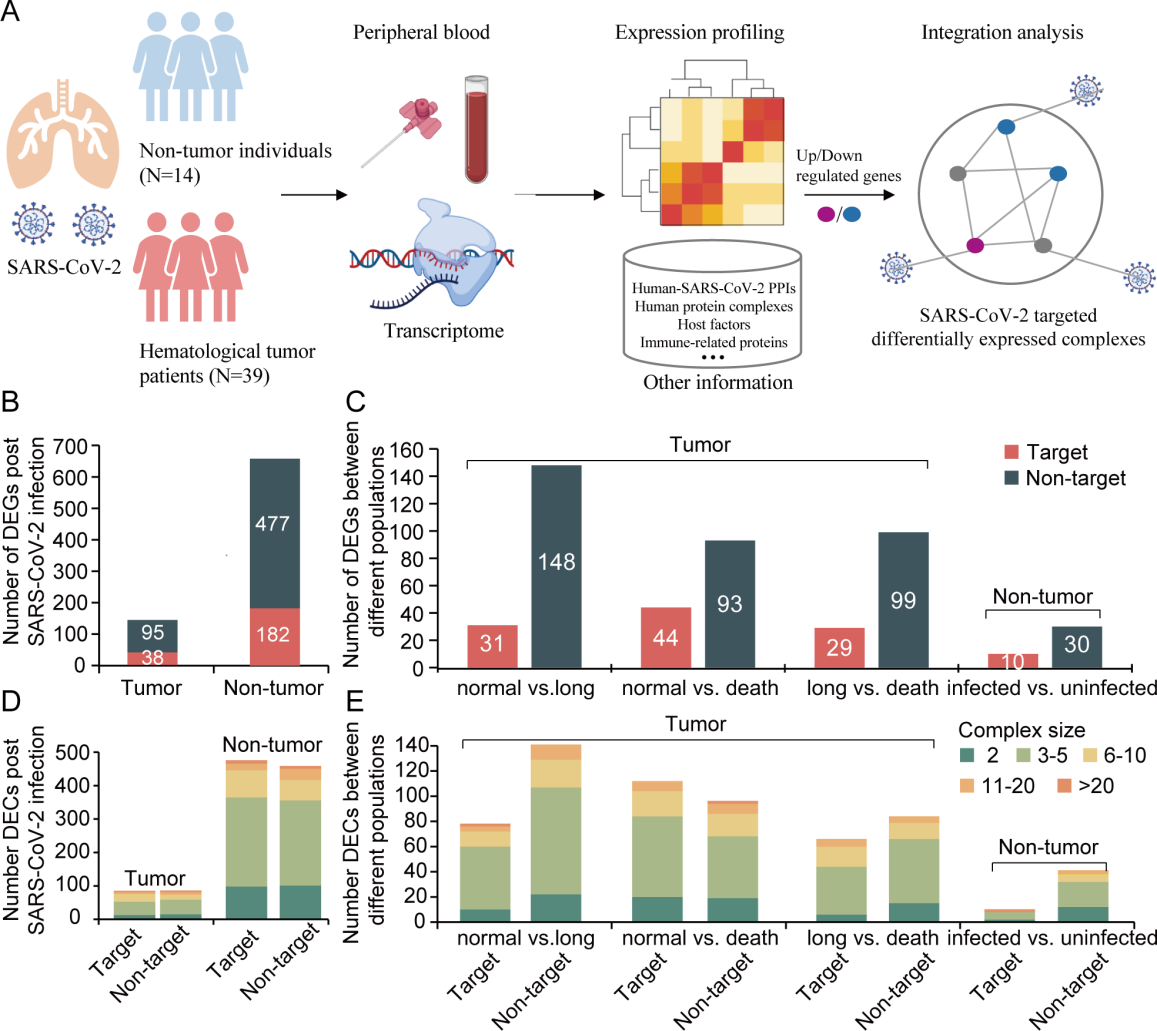
Multi-stage transcriptome atlas of COVID-19 patients with and without hematological tumors. (**A**) Flowchart of the overall experimental design. (**B**) Distribution of differentially expressed viral targets and nontargets of COVID-19 patients with (hematological tumors patients) and without (non-tumor individuals) hematological tumors post-SARS-CoV-2 infection. (**C**) Distribution of differentially expressed targets and nontargets of COVID-19 patients with hematological tumors (normal infection, long infection, and acute death) and uninfected non-tumor individuals (ever infected vs ever uninfected). (**D**) Distribution of targets and nontargets in differentially expressed complexes of COVID-19 patients with (hematological tumors patients) and without (non-tumor individuals) hematological tumors post-SARS-CoV-2 infection. (**E**) Distribution of targets and nontargets in differentially expressed complexes of COVID-19 patients with hematological tumors (normal infection, long infection, and acute death) and distribution of targets and nontargets in differentially expressed complexes of uninfected non-tumor individuals (ever infected vs ever uninfected).

A total of 39 hematological tumor patients with COVID-19 and 14 non-tumor individuals (Department of Hematology, Peking University First Hospital) provided 94 samples from peripheral whole blood that were bulk RNA sequenced (Fig. 1A). Hematological tumor and non-tumor samples were further divided into pre-infection (*N* = 13), mid-infection (*N* = 47), and post-infection convalescence (*N* = 34) according to the timeline of SARS-CoV-2 infection. Samples of hematological tumor patients were also categorized into normal (*N* = 50), persistent long infection (*N* = 11), and acute death (*N* = 7) groups according to the outcome of SARS-CoV-2 infection. Furthermore, samples of non-tumor individuals were binned into infected (*N* = 22) and uninfected (*N* = 5) groups. As for the latter, the number of uninfected samples was limited as most non-tumor individuals were also infected during the outbreak of SARS-CoV-2. As for the definition of long COVID, we complied with the United Kingdom (UK) National Institute for Health and Care Excellence guidelines that defined long COVID as persistence of symptoms beyond 4 weeks of SARS-CoV-2 infection (21, 22) and considered acute death as patients who deceased within 2 weeks post-infection. Furthermore, we collected corresponding clinical information from these hematological tumor patients, including sex, age, diagnosis, symptoms, anti-viral treatment, and 16 biomedical parameters of different COVID-19 stages (Table S1).

### RNA extraction and sequencing

We collected 94 peripheral whole blood samples of hematological tumor patients and non-tumor individuals. Subsequently, the collected samples were mixed with trizol and then flash frozen at −80°C. We extracted total RNA using the Standard Sensitivity RNA Analysis Kit (15 nt) (DNF-471) (Qiagen) and further removed human globin proteins. RNA library construction and RNA sequencing were performed on the Illumina Platform of Shenzhen BGI Technology. Briefly, after RNA extraction, RNA concentrations were detected using a fluorometric Qubit RNA assay, and the integrity of the RNA was determined on an Agilent 2100 Bioanalyzer (Agilent Technologies) according to the manufacturer’s protocols. Furthermore, we removed the globin mRNA by using the GLOBINclear Kit. To construct the libraries, high-quality total RNA samples (RIN ≥ 7) were processed using NEBNext Ultra RNA Library Prep Kit, E7530S according to the manufacturer’s protocols. Briefly, we purified mRNA molecules from total RNA using oligo(dT)-attached magnetic beads, which were fragmented and reverse-transcribed using random primers. Libraries were quantified by qPCR, and library profiles were assessed using the DNA High Sensitivity LabChip kit on an Agilent Bioanalyzer. Libraries were sequenced on the Illumina HiSeq 2500 platform.

### RNA-Seq data processing and analysis

Clean data were obtained after routine filtering (i.e., removing adapter sequences and low-quality reads) by SOAPnuke (23). Clean reads of each sample were aligned to the human GRCh38 genome (https://www.ncbi.nlm.nih.gov/assembly/GCF_000001405.39) through HISAT2 (24). Subsequently, we employed StringTie (25) to assemble the transcriptome of each sample and determined the expression of all genes using RSEM (26). In particular, we determined differentially expressed genes (DEGs) between different populations such as non-tumor (ever infected and uninfected), hematological tumor (normal infection, long infection, and acute death), or different time points (pre-infection, mid-infection, and post-infection convalescence) through the R Bioconductor package “DESeq2” (27). Specifically, we considered genes as differentially expressed when |log_2_FC| was ≥1 and the FDR-adjusted *P*-value was ≤0.05. Read counts were also normalized to transcripts per million through StringTie v2.8.4 to show gene expression abundance. All genes were represented by their corresponding gene names.

### Human-SARS-CoV-2 PPIs

We first downloaded experimentally determined human-SARS-CoV-2 protein-protein interactions (PPIs) from BioGRID (15) and further removed redundant interaction pairs according to the gene names of protein pairs. Consequently, we obtained a total of 23,871 non-redundant human-SARS-CoV-2 PPIs between 31 SARS-CoV-2 proteins and 6,442 human proteins.

### Human protein complexes and PPIs

A total of 5,204 and 6,965 human protein complexes were originally collected from CORUM (18) (https://mips.helmholtz-muenchen.de/corum/) and hu.MAP (19) (http://proteincomplexes.org), respectively. Furthermore, experimentally determined human PPIs were collected from BioGRID (15). After removing redundancy and non-physical interactions, we obtained 80,258 human PPIs between 11,141 human proteins. Subsequently, we mapped these human PPIs on our collected complexes and removed redundant complexes with the same subunits as well as complexes without any human PPIs, allowing us to obtain a total of 9,475 human protein complexes.

### Essential genes, scaffold proteins, SARS-CoV-2’s host factors, and human innate immune-related proteins

To characterize the functional roles of differentially expressed genes including SARS-CoV-2 (non-)targets i.e., (non-)interacting human proteins of SARS-CoV-2 in hematological tumor patients and non-tumor individuals, we focused on four functional genes/proteins capturing essential genes, scaffold proteins, SARS-CoV-2’s host factors and human immune-related proteins. First, we collected 5,169 human essential genes that were indispensable for the human host in diverse cellular processes from references 28, 29. Furthermore, we collected 273 scaffold proteins from ScaPD (30), 299 SARS-CoV-2’s host factors from three large-scale CRISPR screening studies (31–33), and 1,044 human innate immune-related proteins from InnateDB (34). Scaffold proteins play important roles in a variety of cellular signaling pathways due to their ability to promote complex assembly. Host factors include two types, such as host dependency factors and host restriction factors, that facilitate or protect against viral infection, respectively. Innate immune-related proteins not only play a critical role in the first line of defense against viral invasion but are also related to the regulation and formation of subsequent adaptive immune responses.

### Network topological and functional enrichment analysis

To characterize network patterns of DEGs post-SARS-CoV-2 infection, we calculated the number of interaction partners of DEGs in the human PPI network (i.e., degree) as well as the corresponding within-complex degree by using the R package “igraph” (https://igraph.org/). As for targeted DEGs, we employed Cytoscape to visualize the human-SARS-CoV-2 PPI network between SARS-CoV-2 proteins and 38 unique/shared DEGs of hematological tumor patients compared to non-tumor individuals.

To find functional and pathway enrichments of identified or targeted DEGs, we downloaded the Gene Ontology (GO) annotation data of human proteins from http://current.geneontology.org/ (35). Moreover, KEGG pathway data were downloaded from https://www.genome.jp/kegg/ (36). Using all human proteins mapped to Cellular Component, Biological Process, and Molecular Function ontologies as well as all human proteins in all the KEGG pathways as reference sets, enriched GO terms and KEGG pathways for the DEGs were determined by hypergeometric tests and deemed significant if corresponding Benjamin-Hochberg corrected *P-*values were ≤0.05.

## RESULTS

### The landscape of differentially expressed genes and complexes post-SARS-CoV-2 infection

Utilizing our transcriptome sequencing data, we identified 133 and 659 DEGs between post-infection convalescence and mid-infection in hematological tumor patients and non-tumor individuals, respectively (Fig. 1B). Moreover, a total of 38 and 182 DEGs interacted with proteins of SARS-CoV-2. We also performed differential expression analysis between different populations of hematological tumor patients (infection cases: normal infection vs long infection, normal infection vs death cases, and long infection vs death cases) and non-tumor individuals (uninfected cases: ever infected vs never infected) (Fig. 1C). Considering all comparisons, we found more DEGs that were not directly targeted by proteins of SARS-CoV-2. Furthermore, we collected 9,475 non-redundant human protein complexes from CORUM and hu.MAP databases and 6,442 human-SARS-CoV-2 PPIs from BioGRID to obtain differentially expressed complexes (DECs) and virally targeted DECs (VTDECs) to analyze SARS-CoV-2 infection mechanisms and their complex-specific difference in hematological tumor patients and non-tumor individuals. Specifically, a protein complex containing at least one DEG was defined as a DEC. Moreover, VTDECs were defined as DECs that contained at least one subunit that interacted with SARS-CoV-2 proteins. Notably, we obtained a total of 166 DECs of hematological tumor patients and 904 DECs in non-tumor individuals, respectively, while 83 and 476 DECs were targeted by SARS-CoV-2 (also called VTDEC) (Fig. 1D). Investigating the frequencies of DECs of different sizes, we found that most DECs had three to five proteins, an observation that held for all different populations (Fig. 1E).

### Dynamic transcriptomic analysis of non-tumor individuals and hematological tumor patients with SARS-CoV-2 infection

**Fig 2:**
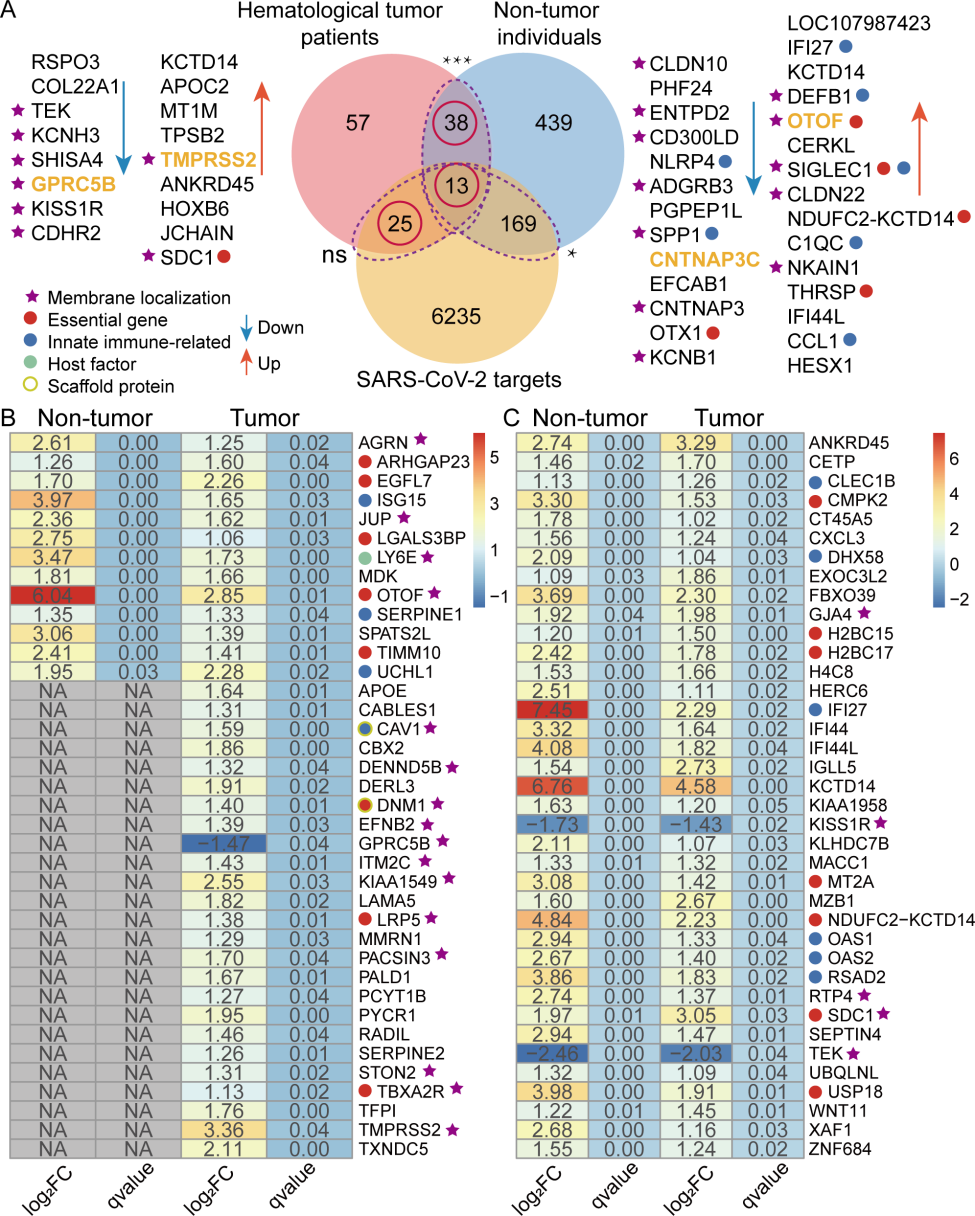
Transcriptome changes in COVID-19 patients with and without hematological tumors. (**A**) Overlapping and unique genes comparing DEGs of hematological tumor patients and non-tumor individuals post-SARS-CoV-2 infection and corresponding distribution of SARS-CoV-2 targets/nontargets. The gene lists in the left and right panels indicate the top DEGs (ordered by |log_2_FoldChange| from largest to smallest) of hematological tumor patients and non-tumor individuals, respectively, where yellow fonts represent SARS-CoV-2 targets. DEGs of non-tumor individuals significantly overlap with DEGs of hematological tumor patients (***P-value < 0.001; two-sided Fisher’s exact test) and SARS-CoV-2 targets (*P-value < 0.05; two-sided Fisher’s exact test). A total of 13 targets in panel **B** and 38 nontargets in panel **C** were shared DEGs between hematological tumor patients and non-tumor individuals, while a total of 25 targets in panel **B** and 169 targets of DEGs were unique for hematological tumor patients and non-tumor individuals, respectively. Most targeted genes in panel **B** were membrane-localized, essential, innate immune-related, host factors, or scaffolding proteins compared to non-targeted genes in panel **C**.

Focusing on dynamic transcriptome changes post-SARS-CoV-2 infection, we identified 133 DEGs (125 upregulated and 8 downregulated) and 659 DEGs (515 upregulated and 144 downregulated) when we compared post-infection convalescence and mid-infection in hematological tumor patients and non-tumor individuals, respectively (Fig. 2A). The number of DEGs in hematological tumor patients was obviously lower than in non-tumor individuals, which is potentially due to the fact that the expression of some genes in tumor patients has not returned to normal levels (i.e., the infection impact caused by SARS-CoV-2 invasion has not been completely cleared in tumor patients). In particular, there were no DEGs in non-tumor individuals between pre-infection and post-infection convalescence, suggesting that non-tumor individuals rapidly clear SARS-CoV-2 compared to patients with hematological tumors. Furthermore, we identified 38 and 182 SARS-CoV-2 targets in DEGs when we compared hematological tumor patients and non-tumor individuals, respectively, based on the experimentally determined high-confidence human-SARS-CoV-2 protein-protein interactions. We found a total of 13 genes that were differentially expressed post-SARS-CoV-2 infection in both hematological tumor patients and non-tumor individuals, representing a uniformly upregulated trend (Fig. 2A and B). In particular, out of the 13 DEGs, LY6E was a host restriction factor that interacts with the SARS-CoV-2 protein NSP6 to limit the entry of SARS-CoV-2 (7, 37). Other 25 DEGs were unique SARS-CoV-2 targets in hematological tumor patients, where the only downregulated gene GPRC5B was a G-protein coupled receptor localized in cell membranes (Fig. 2A and B). In addition, a total of 38 genes were differentially expressed in both populations that were not known SARS-CoV-2 targets, most of which were involved in the antiviral immune response and cytokine-mediated signaling pathways (Fig. 2A and C). Such observations suggest that these genes may be potential SARS-CoV-2 targets or indirectly involved in the regulation pathways that are associated with SARS-CoV-2 infection.

### Network topological and functional role analysis of DEGs and DECs

**Fig 3:**
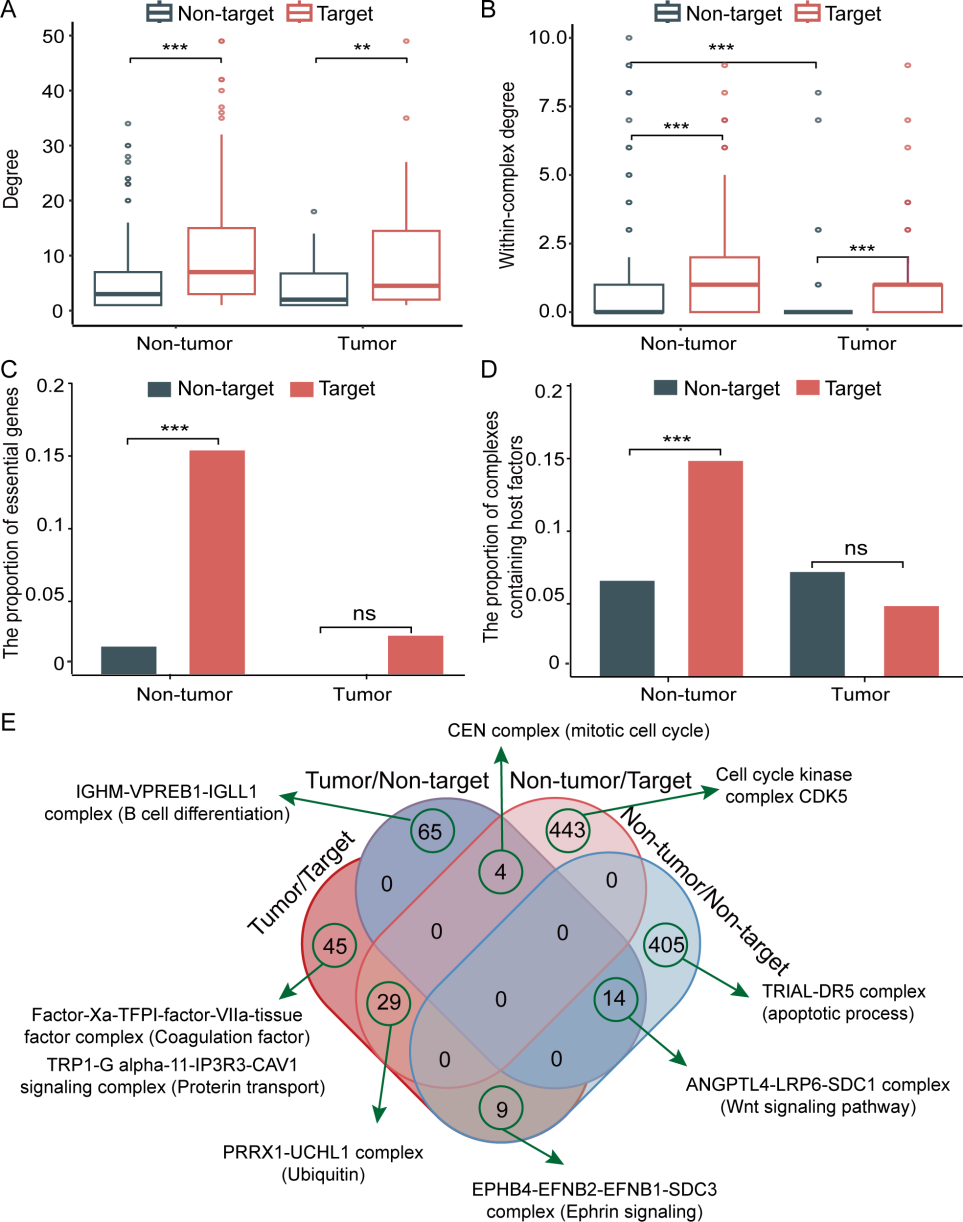
Comparison of network and functional characteristics between SARS-CoV-2 targets and nontargets of hematological tumor patients/non-tumor individuals within DEGs and DECs. (**A**) Degree distributions of DEGs. (**B**) Within-complex degree distributions of DEGs. (**C**) Proportions of essential genes. (**D**) Proportions of complexes containing host factors. (**E**) Specific complex distribution and complex cases of targets/nontargets in DEGs of hematological tumor patients/non-tumor individuals post-SARS-CoV-2 infection.

As for their network characteristics, viruses usually manipulate human host cells by targeting hub proteins (38). To examine the relevant network topological patterns of DEGs in both whole host PPI networks and subnetworks such as DECs and VTDECs, we collected 80,258 experimentally determined high-confidence human PPIs and further mapped them to DECs and VTDECs. Moreover, we calculated the interaction partners of each DEG (i.e., degree) in the human PPI network and the within-complex degree of each subunit in the DECs and VTDECs. We observed that the degree of targets of hematological tumor DEGs post-SARS-CoV-2 infection was significantly higher compared to nontargets (Wilcoxon rank sum test, *P*-value < 0.01; Fig. 3A), an observation that we made in non-tumor individuals as well (*P*-value < 0.001; Fig. 3A). In particular, we found that the within-complex degree of subunits of targeted DECs was significantly higher compared to DECs that were not targeted by SARS-CoV-2 (Wilcoxon rank sum test, *P*-value < 0.001; Fig. 3B). Moreover, the degree of subunits of DECs without SARS-CoV-2 targets in hematological tumors was significantly lower compared to non-tumor individuals (*P*-value < 0.001; Fig. 3B).

We also investigated the functional properties of DEGs and DECs of hematological tumor patients and non-tumor individuals at both the gene and complex levels. Specifically, we focused on four functional proteins, such as scaffold proteins, essential proteins, host factors, and innate immune-related proteins. We observed that the proportion of essential genes of targeted DEGs post-SAR-CoV-2 infection in non-tumor individuals was significantly higher compared to non-targeted DEGs but did not find any significant difference in hematological tumor patients (*P*-value > 0.05, two-sided Fisher’s exact test; Fig. 3C). On a complex level, we found that the proportion of targeted DECs with host factors was significantly higher compared to non-targeted DECs in non-tumor individuals, while there was no significant difference in hematological tumor patients (*P*-value > 0.05, two-sided Fisher’s exact test; Fig. 3D).

Essential genes and host restriction factors are important for the human host to maintain self-life activity and resist viral infection, while, in turn, host dependency factors help viruses to invade host cells. Such observations suggest that SARS-CoV-2 may be more inclined to directly target these important factors to infect and regulate human host cells in non-tumor individuals than hematological tumor patients. Focusing on targeted and non-targeted DECs of hematological tumor patients and non-tumor individuals (Fig. 3E; Table S2), we found that targeted DECs (e.g., PRRX1-UCHL1 complex and CEN complex) were usually enriched with functions/pathways such as ubiquitin and cell cycle (Fig. 3E). Interestingly, several unique DECs of hematological tumor patients post-SARS-CoV-2 infection were associated with blood coagulation and B cell differentiation (Fig. 3E). Moreover, CAV1 as a unique upregulated DEG in hematological tumors may act as a scaffolding protein within caveolar membranes (39) and is involved in the costimulatory signal essential for T-cell receptor-mediated T-cell activation. The binding of CAV1 to DPP4 induces T-cell proliferation and NF-kappa-B activation in a T-cell receptor/CD3-dependent manner (40). In particular, CAV1 negatively regulates TGFB1-mediated activation of SMAD2/3 by mediating the internalization of TGFBR1 from membrane rafts leading to its subsequent degradation (41). TGFB1 and SMAD3 are innate immune-related proteins, while CAV1 is localized to the membrane, suggesting that CAV1 may be a potential target specific to hematological tumor patients with SARS-CoV-2 infection.

### Functional enrichment analysis of DEGs

**Fig 4:**
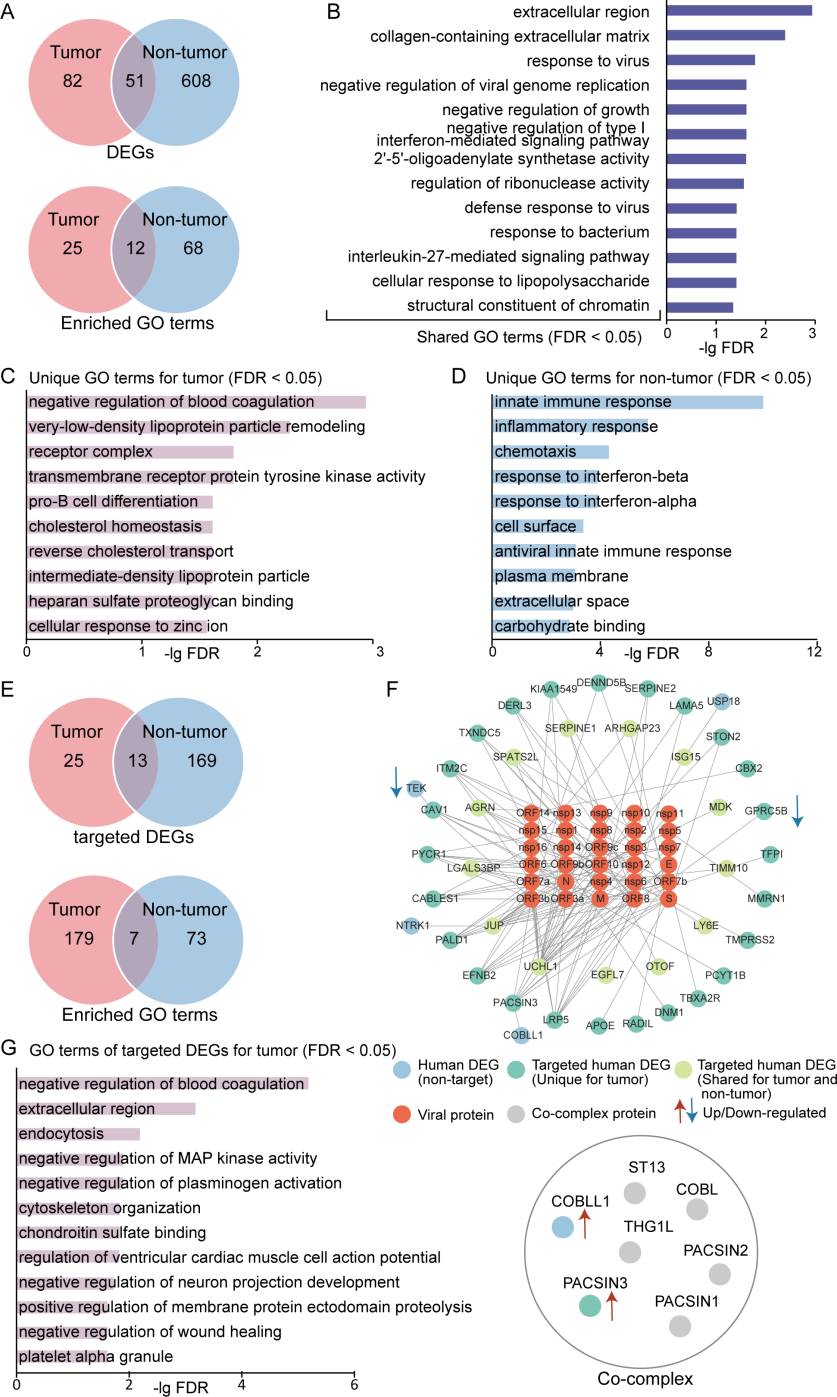
Functional enrichments of DEGs and SARS-CoV-2-targeted DEGs of COVID-19 patients with (hematological tumor patients) and without hematological tumors (non-tumor individuals) post-SARS-CoV-2 infection. (**A**) Distribution of DEGs and their corresponding enriched GO terms. (**B**) Significantly enriched GO terms that were shared between DEGs of hematological tumor patients and non-tumor individuals. (**C**) Significantly enriched GO terms that were unique for DEGs of hematological tumor patients. (**D**) Significantly enriched GO terms that were unique for DEGs of non-tumor individuals. (**E**) Distribution of SARS-CoV-2-targeted DEGs and their corresponding enriched GO terms. (**F**) Protein interaction network between SARS-CoV-2 proteins and human DEGs post-SARS-CoV-2 infection of hematological tumor patients and an upregulated co-complex case. (**G**) Significantly enriched GO terms that were unique for SARS-CoV-2-targeted DEGs of hematological tumor patients.

To understand and compare which host functions are directly or indirectly perturbed by SARS-CoV-2 in hematological tumor patients and non-tumor individuals, we investigated SARS-CoV-2-targeted functions/pathways through a functional GO enrichment analysis. Broad functions that were enriched with host proteins included immune response, viral process, and interferon signaling pathway (Fig. 4). Focusing on the two populations post-SARS-CoV-2 infection, we found 37 and 80 GO terms that were enriched in 133 DEGs of hematological tumor patients and 659 DEGs of non-tumor individuals, respectively (Fig. 4A; Table S3). In turn, we obtained a total of 186 and 80 enriched GO terms when we considered 38 and 182 SARS-CoV-2-targeted DEGs in hematological tumor patients and non-tumor individuals, respectively (Fig. 4E; Table S4). Interestingly, the number of enriched GO terms of DEGs in hematological tumor patients was much smaller than in non-tumor individuals (Fig. 4A), while the number of enriched GO terms in SARS-CoV-2-targeted DEGs showed an opposite trend (Fig. 4E). Such findings suggest that differentially expressed targets in hematological tumor patients post-SARS-CoV-2 infection were enriched with more biological functions than nontargets and targets/nontargets in non-tumor individuals, potentially reflecting a stronger host immune response. Furthermore, we found 13 commonly shared GO terms in DEGs of hematological tumor patients and DEGs of non-tumor individuals, including response to bacteria, response to viruses, negative regulation of viral genome replication, negative regulation of growth, and negative regulation of type I interferon-mediated signaling pathway (Fig. 4B). In particular, significantly enriched GO terms of DEGs of hematological tumor patients mainly revolved around negative regulation of blood coagulation (APOE, SERPINE1, SERPINE2, and TFPI), very-low-density lipoprotein particle remodeling (APOC2, APOE, and CETP), and pro-B cell differentiation (FLT3 and SOX4) (Fig. 4C). Such observations indicate that SARS-CoV-2 infection had greater potential effects on coagulation, lipoprotein remodeling, and B cell differentiation in patients with hematological tumors compared to non-tumor individuals. In particular, APOE is an unique upregulated DEG of hematological tumor patients, which affects both blood coagulation and lipoprotein remodeling. APOE interacts with both the SARS-CoV-2 Spike protein and human host ACE2 protein. Chen et al. (42) demonstrated that APOE was associated with severe COVID-19 outcomes through the downregulation of ACE2 and imbalanced RAS pathway. Another study showed that APOE interacts with ACE2, inhibiting SARS-CoV-2 cellular entry and inflammation in COVID-19 patients (43). Such observations imply that APOE plays an important role in SARS-CoV-2 infection, while APOE has been identified as a clinical trial drug target for familial hypercholesterolemia and hyperlipidemia as of the TTD database (44) (https://db.idrblab.net/ttd). Given its upregulation in patients with hematological tumors, APOE may be a potentially valuable specific drug target for COVID-19 patients with hematological tumors.

The unique enriched GO terms of DEGs of non-tumor individuals were mostly related to shared GO terms such as innate immune response, response to interferon, and anti-viral response (Fig. 4D). Regarding targeted DEGs, we mainly focused on hematological tumor cases that allowed us to determine 25 unique targets and 13 shared targets in DEGs post-SARS-CoV-2 infection in comparison to non-tumor individuals (Fig. 4E and F). Out of the top 10 unique enriched GO terms, the most significantly enriched term (i.e., negative regulation of blood coagulation) was also most significantly enriched in all DEGs including both targets and nontargets of hematological tumor patients (Fig. 4G). In addition, the enriched terms such as negative regulation wound healing and platelet alpha granule complemented the details of the effect of targets on the function of blood in patients with hematological tumors (Fig. 4G). Focusing on the human-SARS-CoV-2 interaction network of DEGs of hematological tumor patients, we observed four non-targeted DEGs (TEK, USP18, NTRK1, and COBLL1) that interact with known SARS-CoV-2 targets by mapping human PPIs to this network (Fig. 4F). We further investigated complexes that contained these proteins and corresponding targets and found that COBLL1 and PACSIN3 were localized in the same complex (Fig. 4F). In particular, the observed complex relates to the reorganization of the actin cytoskeleton, the negative regulation of endocytosis, calcium ion transport, and tRNA processing. Such observations suggest that SARS-CoV-2 infection may result in the consistent upregulation of interacting PACSIN3 and COBLL1 to affect endocytosis, calcium ion transport, and tRNA processing.

### Comparative analysis of transcriptomes, complexes, and clinical indicators in different clinical infection types of hematological tumor patients

**Fig 5:**
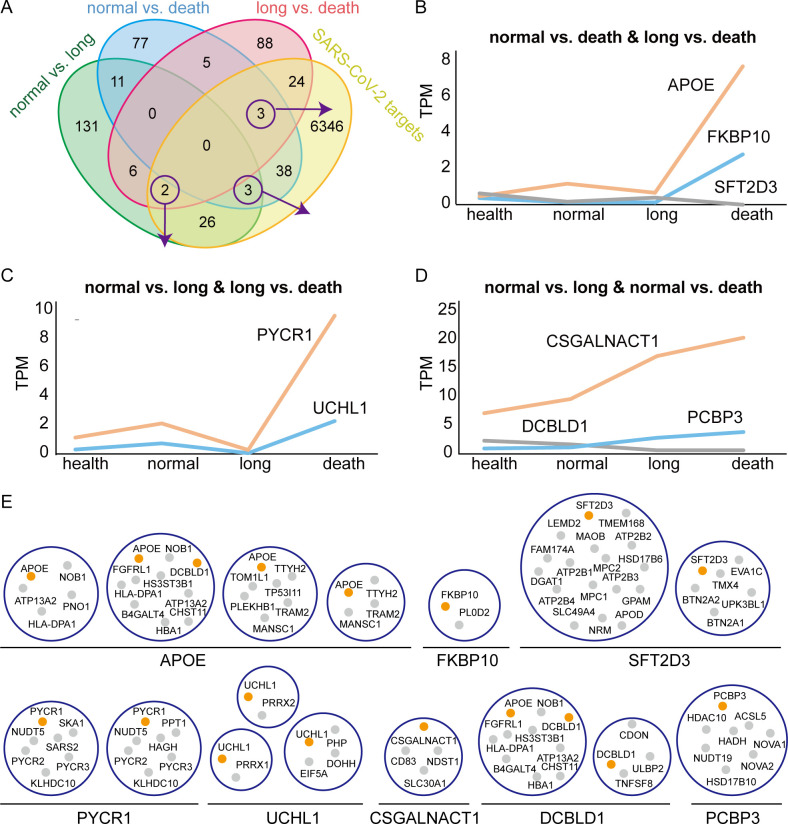
DEGs and DECs between multiple infection statuses of hematological tumor patients. (**A**) Distribution of DEGs between multiple infection statuses, including normal infection, long infection, and acute death of hematological tumor patients and their overlaps with SARS-CoV-2 targets. (**B–D**) Expression atlas of shared targeted DEGs between any two treatment-control groups. (**E**) Specific DECs of DEGs in panels **B–D**.

To explore the infection mechanism difference of hematological tumor patients with different clinical phenotypes, we investigated the transcriptome changes comparing these infection groups (i.e., normal infection, long infection, and acute death). We first identified 179, 137, and 128 DEGs between any two groups (i.e., “normal vs long,” “normal vs death” and “long vs death”), respectively (Fig. 5A). Subsequently, we mapped experimentally verified SARS-CoV-2 targets onto these genes and obtained 31, 44, and 29 targeted DEGs, respectively (Fig. 5A). These DEGs were enriched with GO terms, such as “cell surface receptor signal pathway,” “B cell differentiation,” “primary immunodeficiency,” “cytokine receptors,” and “lipoprotein particle clearance” (Tables S5 and S6), which were similar to the uniquely enriched terms of DEGs when we compared post-infection convalescence and mid-infection of hematological tumor patients. Furthermore, 217, 207, and 150 DECs were obtained by integrating human complex data, respectively, while 77, 112, and 66 of them were targeted by SARS-CoV-2 (Table S7). We mainly focused on targeted genes and complexes that were differentially expressed in more than two comparative groups. For example, APOE, FKBP10, and SFT2D3 were differential genes in both “normal vs death” and “long vs death” cases (Fig. 5B through E). Specifically, we found that the expression of APOE was significantly upregulated in the acute death cases compared to both normal and long infection cases during SARS-CoV-2 infection. Notably, APOE was also differentially expressed in post-infection convalescence and mid-infection of hematological tumor patients but not in non-tumor individuals (Fig. 2B). Such findings indicate that APOE plays a crucial role in SARS-CoV-2 infection of patients with hematological tumors and potentially affects the survival prognosis of patients, especially showing higher levels of expression in long infection and dead patients. Interestingly, we found that APOE and DCBLD1 occurred in the same complex (subunits: APOE, ATP13A2, B4GALT4, CHST11, DCBLD1, FGFRL1, HBA1, HLA-DPA1, HS3ST3B1, and NOB1; Fig. 5E), which were enriched with biological processes such as “peptide antigen assembly with MHC class II protein complex,” “cellular oxidant detoxification,” “intracellular calcium ion homeostasis,” “blood microparticle,” “virion assembly,” and “lipid transport.” In particular, DCBLD1 contains three functional domains, i.e., CUB, LCCL, and coagulation factor 5/8 C-terminal domains (45–47), which are associated with tissue repair, axon guidance and angiogenesis, inflammation, receptor-mediated endocytosis, bacterium infection, tumor suppression, and blood coagulation. Moreover, DCBLD1 interacts with KRAS, which is a key diagnostic and prognostic gene associated with various hematological tumors such as juvenile myelomonocytic leukemia (48, 49). In contrast to APOE, DCBLD1 was significantly downregulated in acute death cases compared to both normal and long infection cases during SARS-CoV-2 infection. In addition, DCBLD1 was not significantly differentially expressed between post-infection convalescence and mid-infection of patients, suggesting that DCBLD1 was indirectly affected by SARS-CoV-2 infection and further resulted in different prognosis outcomes in different patients with hematological tumors. Considering the characterization of membrane localization (50), DCBLD1 is potentially a valuable drug target for further studies.

**Fig 6:**
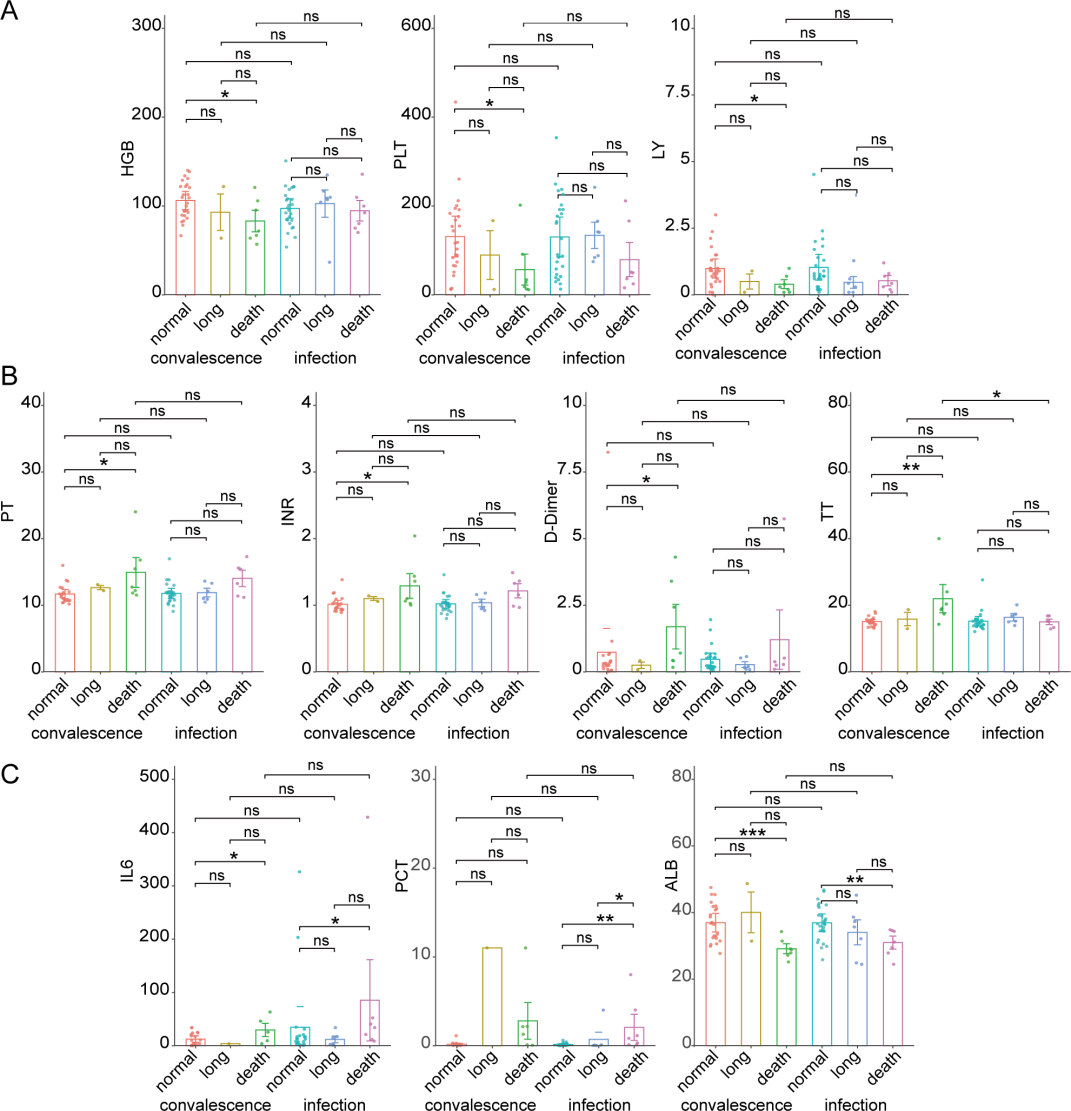
Significantly different clinical indicators between death and normal/long infection cases. (**A**) Three blood indicators: HGB, PLT, and LY. (**B**) Four coagulation indicators: PT, INR, and TT. (**C**) Three immune-related indicators: IL6, PCT, and ALB. *, **, and *** indicate *P*-values < 0.05, 0.01, and 0.001 (Wilcoxon rank sum test), respectively.

Focusing on our collected 16 clinical parameters of these hematological tumor patients (“normal,” “long,” and “death”) during infection and post-infection convalescence (Table S1), we found 10 significantly different parameters between normal infection and deceased patients (Fig. 6). Specifically, three blood indicators, i.e., hemoglobin (HGB), platelets (PLT), and lymphocytes (LY) of death cases were significantly lower compared to normal infection cases during convalescence (Fig. 6A), while four coagulation indicators, i.e., prothrombin time (PT), international normalized ratio (INR), D-Dimer, and thrombin time (TT) showed significantly higher values in death cases than in normal infection cases (Fig. 6B), indicating weaker immunity and higher risk of bleeding. In particular, the levels of three immune-related indicators, i.e., interleukin 6 (IL6), procalcitonin (PCT), and albumin (ALB), were significantly different during infection; the IL6 levels of death cases were significantly greater than in normal infection, suggesting more inflammatory and immune responses. Larger PCT in death cases compared to normal and long infection, as well as lower ALB, reveals a state of severe infection from which recovery is difficult (Fig. 6C). Such observations were consistent with our findings, which suggests the reliability and potential of our DEGs and DECs.

### Comparative analysis of transcriptomes and complexes in different types of non-tumor individuals

**Fig 7:**
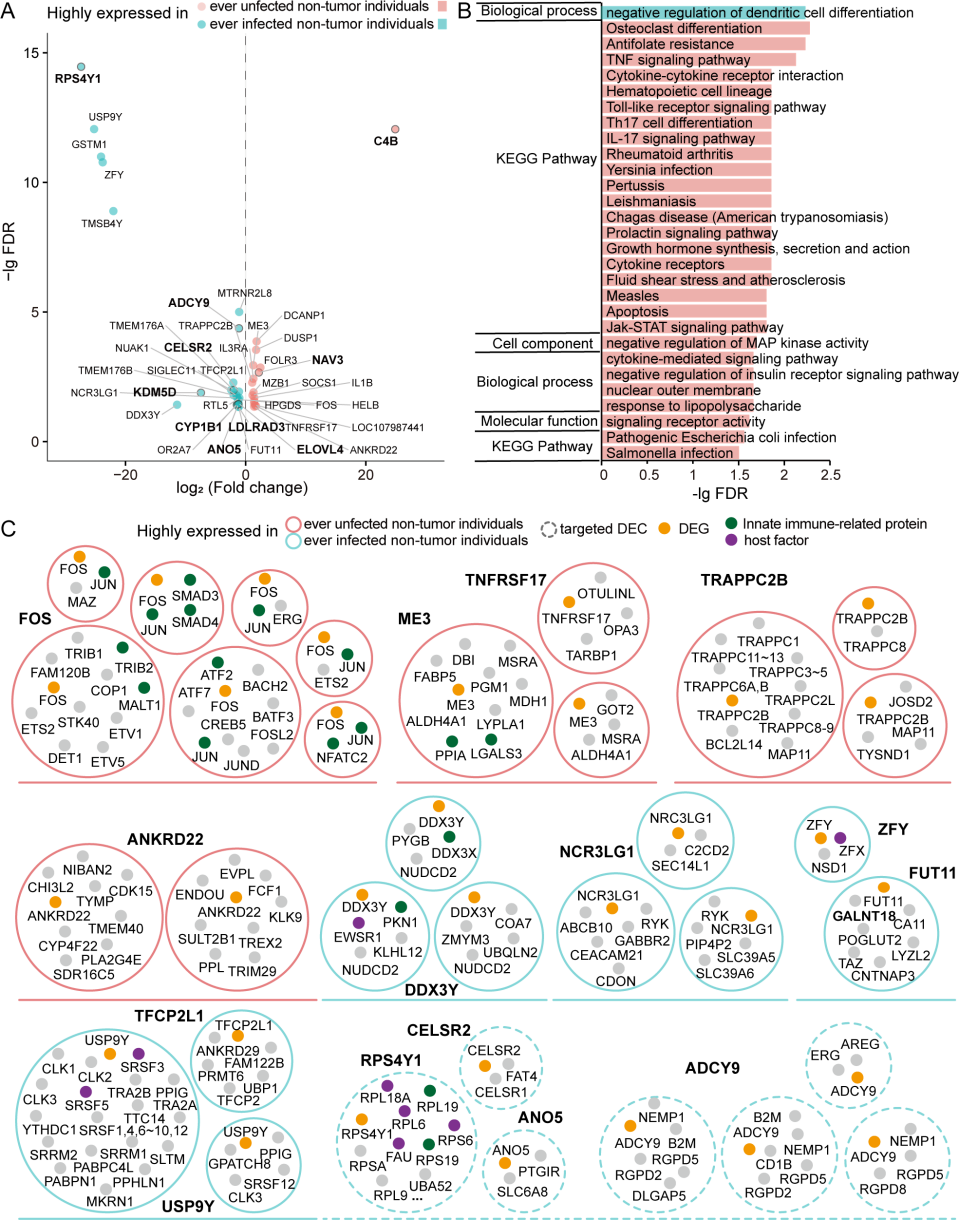
DEGs and DECs between non-tumor individuals ever SARS-CoV-2 infected and uninfected in the 2022 COVID-19 pandemic. (**A**) A total of 40 genes including 10 SARS-CoV-2 targets were identified as DEGs between non-tumor individuals ever SARS-CoV-2 infected and uninfected. (**B**) Significantly enriched functional terms and pathways of these upregulated and downregulated DEGs, respectively. (**C**) Up- and downregulated DECs of these DEGs including targets and nontargets.

To explore the potential factors of susceptibility to SARS-CoV-2 at the transcriptome level, we detected DEGs between uninfected samples of two groups of non-tumor individuals who were ever infected and uninfected in the 2022 SARS-CoV-2 (Omicron BF.7 variant strain) pandemic in Beijing. A total of 16 (upregulated) and 24 (downregulated) genes were significantly expressed in ever uninfected and infected non-tumor individuals, respectively (Fig. 7A). Out of these DEGs, two upregulated (i.e., C4B and NAV3) and eight downregulated genes (i.e., RPS4Y1, ADCY9, CELSR2, KDM5D, CYP1B1, ANO5, LDLRAD3, and ELOVL4) were also SARS-CoV-2 targets (Fig. 7A). We further investigated the functional roles of these up- and downregulated genes and observed that genes significantly highly expressed in ever infected non-tumor individuals (i.e., 24 downregulated genes) were enriched only with negative regulation of dendritic cell differentiation (Fig. 7B; Table S8). Genes that we found highly expressed in ever uninfected non-tumor individuals (i.e., 16 upregulated genes) were enriched in functions/pathways such as tumor necrosis factor (TNF) signaling pathway, cytokine-cytokine receptor interaction, hematopoietic cell lineage, and apoptosis (Fig. 7B; Table S8). In particular, the most significantly highly expressed gene C4B (Complement component 4B) is essential for the propagation of the classical complement pathway and is a known innate immune-related protein as found in the InnateDB (https://www.innatedb.ca/). Specifically, C4B covalently binds to immunoglobulins and immune complexes and enhances the solubilization of immune aggregates and the clearance of IC through CR1 in erythrocytes. Such observations suggest that patients with high levels of C4B expression may be less likely to be infected with SARS-CoV-2 as a consequence of their enhanced immunity.

Moreover, these DEGs were involved in 49 complexes in total, while nine complexes were targeted by SARS-CoV-2 (Table S9). We further removed smaller complexes that completely overlapped with the largest one and thus obtained 26 DECs and 7 targeted DECs (Fig. 7C). In particular, FOS as a subunit of the multimeric SMAD3/SMAD4/JUN/FOS was highly expressed in ever uninfected non-tumor individuals by SARS-CoV-2, a complex that participates in biological process/pathways, such as positive regulation of transcription, DNA binding, and transforming growth factor beta receptor signaling pathway. Specifically, FOS as a nuclear phosphoprotein forms a tight but non-covalently linked complex with the transcription factor AP-1 subunit JUN (51). During TGF-beta activation, the SMAD3/SMAD4/JUN/FOS complex is formed at the AP1/SMAD-binding site to regulate TGF-beta-mediated signaling (52). In particular, FOS has a critical function in regulating the development of cells destined to form and maintain the skeleton and plays an important role in signal transduction, cell proliferation, and differentiation. Such findings suggest that people with high levels of expression of FOS may potentially have stronger abilities to facilitate cell differentiation/proliferation through the regulation of this protein complex.

## DISCUSSION

Here, we report RNA-Seq data of 94 clinical samples from 39 COVID-19 patients with hematological tumors, 9 non-tumor individuals with COVID-19, and 5 non-tumor individuals without COVID-19 in China and constructed an information-rich data resource to decipher potential pathogenic mechanism of SARS-CoV-2 and host immune responses of COVID-19 patients with hematological tumors on a transcriptome, protein interaction, and complex level. Our data set covered three types of infection samples such as normal infection, long infection, and acute death of COVID-19 patients with hematological tumors according to patient outcomes, providing fine details of the molecular responses to SARS-CoV-2 infection and new insights in the interpretation of potential factors affecting the outcomes of COVID-19 patients with hematological tumors. Besides, two types of uninfected samples of non-tumor individuals (i.e., SARS-CoV-2 infected vs uninfected) provided new clues for the analysis of susceptible and non-susceptible factors and corresponding mechanisms of SARS-CoV-2 infection.

The comprehensive comparison analysis of SARS-CoV-2 infection in hematological tumor patients and non-tumor individuals identified 51 shared DEGs and 82 unique DEGs in hematological tumor patients, where 13 shared DEGs and 25 unique DEGs interacted with proteins of SARS-CoV-2. Such findings at the transcriptome and interaction level and our annotated information of functional roles/subcellular localizations provided potential generic and hematologic tumor-specific therapeutic targets for COVID-19. Focusing on protein complexes, we identified DECs in general and SARS-CoV-2 targeted DECs in particular and investigated the network patterns and functional roles of these genes at both the host PPI network and protein complex level. In general, SARS-CoV-2-targeted DEGs shared higher numbers of interactions in the underlying human PPI network and protein complexes compared to non-targeted DEGs. In particular, non-targeted DEGs of hematological tumor patients held lower degrees compared to non-tumor individuals, suggesting a more indirect regulatory network pattern of SARS-CoV-2 attacks and responses to infection in patients with hematological tumors. Moreover, we also found that the proportion of essential genes among targeted DEGs and the proportion of targeted DECs containing host factors were both significantly higher than among non-targeted DEGs and DECs in non-tumor individuals, respectively, while we found no significant difference in hematological tumor patients. Such observations suggest that DECs and DEGs of hematological tumor patients were directly targeted or indirectly affected by SARS-CoV-2 and generally played comparable functional roles.

In our attempt to dissect the SARS-CoV-2 infection mechanism and its difference between hematological tumor patients and non-tumor individuals, we found that DEGs post-SARS-CoV-2 infection were in general significantly enriched with functional terms/pathways such as response to virus, viral genome replication, and the interferon-mediated signaling pathway. Interestingly, we observed several unique biological processes (e.g., negative regulation of blood coagulation, very low-density lipoprotein particle remodeling, and B-cell differentiation) that were significantly enriched in hematological tumor patients. Similar unique functions of hematological tumor patients were also found in DECs, including targeted DEC Factor-Xa-TFPI-factor-Vila-tissue factor complex (coagulation factor) and non-targeted DEC IGHM-VPREB1-IGLL1 complex (B-cell differentiation), suggesting that SARS-CoV-2 has both direct and indirect effects. In particular, APOE is involved in both lipoprotein remodeling and blood coagulation, and APOE was also differentially expressed when we compared SARS-CoV-2-infected samples of hematological tumor patients who suffered from normal infection, long infection, and acute death. In other words, APOE was only differentially expressed in hematological tumor patients with SARS-CoV-2 infection and was significantly highly expressed in acutely deceased patients compared to patients with normal or long infection. Such findings indicated that APOE putatively plays an important role in SARS-CoV-2 infection of hematological tumor patients and significantly affects clinical outcomes. Furthermore, we found that APOE and another downregulated DEG in “normal vs long/normal vs death,” i.e., DCBLD1, were localized in the same complex. Specifically, genes in this complex were found in biological processes such as “peptide antigen assembly with MHC class II protein complex,” “blood microparticle,” “virion assembly,” and “lipid transport.” In particular, DCBLD1 contains coagulation factor 5/8 C-terminal functional domains and interacts with a key diagnostic and prognostic gene, KRAS, which is associated with various hematological tumors. In contrast to APOE, DCBLD1 was significantly downregulated in acute death cases compared to both normal and long infection cases during SARS-CoV-2 infection. Taken together, APOE, DCBLD1, and other subunits in this complex may be potential drug targets for further studies. Other DEGs and DECs that were differentially expressed comparing hematological tumor patients with different clinical outcome types also provide clues for the understanding of SARS-CoV-2 infection mechanism specific for hematological tumors and the development of the potential therapeutic targets.

To explore the factors that are less susceptible to SARS-CoV-2 infection, we also compared the difference between non-tumor individuals who were (not) infected with COVID-19 on both transcriptome and complex levels. We found a total of 16 and 24 genes that were significantly highly expressed in ever uninfected and infected individuals. In particular, these 16 genes that were highly expressed in ever uninfected individuals were mainly enriched in functions/pathways, such as cytokine-cytokine receptor interaction, TNF signal pathway, interleukin cell differentiation, and various pathogenic infection processes, suggesting that these individuals who never suffered from a COVID-19 infection might have stronger immunity such as higher levels of interleukin cell differentiation for anti-viral infection. Moreover, we identified several DECs that were highly expressed in ever uninfected and infected individuals. The former was involved in more innate-immune-related proteins to enhance the innate immune responses for avoiding infection, while the latter contained more host factors to help or restrict viral infection. Besides, we also found several instances including differentially expressed C4B gene and SMAD3/SMAD4/JUN/FOX complex to explore potential possible mechanistic details of how non-tumor individuals who are not susceptible fend off viral infections. Our findings provide tractable angles and clues to further explore these important questions and develop potential drugs to prevent infection.

We anticipate that these findings and the transcriptome resource will stimulate important research toward characterizing new SARS-CoV-2 strains for patients with hematological tumors, understanding the specific pathogenic mechanisms of COVID-19, and developing effective therapeutics and vaccines for current and future pandemics.

## SUPPLEMENTAL MATERIAL

Supplemental Tables msystems.01385-23-s0001.xlsx Tables S1 to S9.

ASM does not own the copyrights to Supplemental Material that may be linked to, or accessed through, an article. The authors have granted ASM a non-exclusive, world-wide license to publish the Supplemental Material files. Please contact the corresponding author directly for reuse.
